# The Role of the Gravitational Field in Generating Electric Potentials in a Double-Membrane System for Concentration Polarization Conditions

**DOI:** 10.3390/membranes13100833

**Published:** 2023-10-17

**Authors:** Kornelia Batko, Izabella Ślęzak-Prochazka, Weronika Sokołowska, Małgorzata Rak, Wiktoria Płonka, Andrzej Ślęzak

**Affiliations:** 1 Institute of Political Science, University of Silesia, 11 Bankowa Str., 40287 Katowice, Poland; 2 Department of Systems Biology and Engineering, Silesian University of Technology, Akademicka 2A, 44100 Gliwice, Poland; izabella.slezak-prochazka@polsl.pl; 3 Biotechnology Centre, Silesian University of Technology, Akademicka 2A, 44100 Gliwice, Poland; werosok960@student.polsl.pl (W.S.); malgrak825@student.polsl.pl (M.R.);; 4 Collegium Medicum, Jan Dlugosz University, 13/15 Armia Krajowa Al, 42200 Częstochowa, Poland; aslezak52@gmail.com

**Keywords:** membrane transport, Kedem–Katchalsky equations, gravielectric effect, hydrodynamic instability, diffusion, convection

## Abstract

Electric potentials referred to as the gravielectric effect ($∆\Psi_{S}$) are generated in a double-membrane system containing identical polymer membranes set in horizontal planes and separating non-homogenous electrolyte solutions. The gravielectric effect depends on the concentration and composition of the solutions and is formed due to the gravitational field breaking the symmetry of membrane complexes/concentration boundary layers formed under concentration polarization conditions. As a part of the Kedem–Katchalsky formalism, a model of ion transport was developed, containing the transport parameters of membranes and solutions and taking into account hydrodynamic (convective) instabilities. The transition from non-convective to convective or vice versa can be controlled by a dimensionless concentration polarization factor or concentration Rayleigh number. Using the original measuring set, the time dependence of the membrane potentials was investigated. For steady states, the $∆\Psi_{S}$ was calculated and then the concentration characteristics of this effect were determined for aqueous solutions of NaCl and ethanol. The results obtained from the calculations based on the mathematical model of the gravitational effect are consistent with the experimental results within a 7% error range. It has been shown that a positive or negative gravielectric effect appeared when a density of the solution in the inter-membrane compartment was higher or lower than the density in the outer compartments. The values of the $∆\Psi_{S}$ were in a range from 0 to 27 mV. It was found that, the lower the concentration of solutions in the outer compartments of the two-membrane system ($C_{0}$), for the same values of $C_{m}/C_{0}$, the higher the $∆\Psi_{S}$, which indicates control properties of the double-membrane system. The considered two-membrane electrochemical system is a source of electromotive force and functions as an electrochemical gravireceptor.

## 1. Introduction

Both biological and synthetic membranes are sensitive to changes in the physicochemical properties of their thermodynamic environment [1,2]. Therefore, the membrane transport of water with solutes can be regulated by concentration, temperature, electric potential and/or mechanical pressure gradients. The gravitational field plays an important role in membrane transport by inducing or eliminating natural (gravitational) convection [3]. The study of membrane transport processes in such systems is important in many areas of science, technology and biomedicine [4,5,6,7]. Examples of terrestrial biomedical applications include controlled drug release systems, membrane dressings to promote healing of chronic wounds, bioreactors for testing strategies to combat bacterial infections using lytic phage applications in combination with established and novel antimicrobial agents, etc. In these systems, the membrane provides a selective barrier to ensure the separation of the phases. This role is performed by polymeric membranes of different structures and compositions, made of polyvinyl chloride, bacterial cellulose, cellulose acetate, etc. In contrast, studies in the space environment have shown that cells exposed to microgravity experience numerous changes such as loss of gravitational convection, hydrodynamic shear or sedimentation [8]. The disruption of tissue formation has also been observed, with consequent impairment of cellular mechanoreceptors that respond to environmental and internal biophysical stresses. Microgravity conditions also play an important role in the development of materials and process technologies [9].

The term concentration polarization (CP) refers to the effects of creating additional concentration gradients of ionic or non-ionic components in the solution areas of an electrolyte and/or non-electrolyte adjacent to the surface of any selective membrane, separating solutions of different concentrations [10,11,12,13]. These areas, called concentration boundary layers (CBLs), are created on both sides of the membrane in both single- and multi-membrane systems [12,14,15,16,17]. CBLs formation is a result of molecular diffusion and leads to a significant reduction in the concentration gradient (osmotic pressure) and, consequently, in membrane transport [11,12]. These processes are reflected in nonlinear concentration characteristics of volume flux, solute flux and membrane potentials [16,17]. When the concentration (density) gradient is antiparallel to the gravity vector, CBLs undergo natural destruction due to the appearance of natural (gravitational) convection [15,16,17,18,19,20,21,22]. 

The transition from the diffusive to the convective state or vice versa is controlled by the concentration Rayleigh number ($R_{c}$) [20]. For sufficiently large solution density gradients, directed antiparallel to the gravity vector, buoyancy forces prevail over viscous forces, causing the convective mixing of solutions. The fluid behavior for large density differences is described by the Navier–Stokes equations in the Boussinesque approximation [18,19,20,21]. For large values of $R_{c}$ ($R_{c}$ ~ 10^6^), turbulent Rayleigh–Benard convection or its variants occurring on horizontal surfaces is widely studied in the context of technological applications. Turbulent natural convection also occurs in the boundary layers of the atmosphere, the oceans and other large bodies of water, as well as in the Earth’s interior [21].

Different types of spatial–temporal structures of hydrodynamic instabilities can be visualized by optical methods such as Mach–Zehnder interferometry, holographic interferometry or Puthhenveettil et al.’s optical methods, etc. [14,19,20,22]. Depending on the type of boundaries of the studied areas with hydrodynamic instabilities (rigid, free or fuzzy surfaces), different values of critical $R_{c}$, above which gravity-induced hydrodynamic instabilities appear, should be considered [21]. Puthenveettil et al. [19,20] studied transport through a horizontally aligned nylon membrane with a regular square-shaped pore structure. By studying the dynamics of turbulent motions, these authors visualized the structure of plumes (plum structures) formed under turbulent convection in the range of Rayleigh numbers satisfying the condition 10^5^ < $R_{c}$ < 10^11^.

The membrane potential that is a consequence of convective destruction of CBLs in a single-membrane electrochemical cell is called the gravielectric effect [16,17,23,24]. This effect is a consequence of diffusion, concentration polarization and the action of gravity [16,17,23]. For the electrochemical cell design, a system was used in which two solutions with different NaCl or KCl solutions were separated by a synthetic polymer membrane. The solutions were connected to Ag/AgCl electrodes using original bridges [16] or immersed directly in the solutions [24]. In the first case, the dependence of the measured electrical potential difference on the distance of the electrodes from the membrane was eliminated. In the second case, the dependence is obvious. These studies have shown, among other things, that the reversal of the mechanical pressure gradient relative to the concentration (density) gradient has a significant effect on the value of the membrane potential difference [24]. In addition, mathematical models of this effect have been developed using the Kedem–Katchalsky formalism [16,17,24].

According to the idea of Curran and McIntosh [25], a two-membrane system consists of two serially connected single-membrane systems containing two membranes with different transport parameters, separating three homogeneous solutions of different concentrations. Such a system is osmotic-diffusion asymmetric under any hydrodynamic conditions, as manifested by a non-zero osmotic pressure difference generating membrane transport. As a special case, if a two-membrane system contains two identical membranes, they separate three solutions with concentrations meeting the condition $C_{l}$ = $C_{r}$ < $C_{m}$ ($C_{l}$, $C_{r}$—concentrations of solutions in the outer compartments, $C_{m}$—concentration of solution in the inter-membrane compartment); this system is osmotic-diffusion symmetric, resulting in the disappearance of membrane transport both under conditions of solution homogeneity and under conditions of concentration polarization. However, changing the membrane orientation from vertical to horizontal and abandoning the assumption of solution homogeneity (no mechanical mixing of solutions) provides a new group of phenomena, the cause of which is the breaking of osmotic-diffusion symmetry by the gravitational field on one of the membranes [15,26,27,28,29,30]. Kargol included pumping the solution vertically upward (against the force of gravity), circulation of the solution as well as asymmetry and amplification of the graviosmotic flux [30]. This symmetry breaking is based on the fact that, depending on the density of the solution contained in the inter-membrane compartment, the CBL system in the vicinity of one of the membranes is in a non-convective state (hydrodynamically stable) and in the vicinity of the other—in a convective state (hydrodynamically unstable). In this type of double-membrane system, membrane transport referred to as graviosmotic transport occurs.

In a previous paper [15], it was shown that, under conditions wherein the concentration Rayleigh number ($R_{c}$) assumes subcritical values (non-convective state) in the solution regions on both sides of the M_l_ and M_r_ membranes, unobstructed molecular diffusion through both the M_l_ and M_r_ membranes occurs. This leads to a reduction in the concentration gradient across each membrane. Osmotic transport disappears, since the CBL/M_l_/CBL and CBL/M_r_/CBL complexes are symmetric. Such a process should also occur in the space environment (under microgravity). Under conditions of Earth’s gravity, when $R_{c}$ assumes supercritical values, depending on the density of the solutions filling the membrane compartment, undisturbed molecular diffusion occurs only through one of the membranes (vertically upwards or vertically downwards). In the surroundings of the second membrane, free convection occurs, which partially reconstructs the concentration gradient on one of the membranes, acting destructively on the CBLs. As a result of this asymmetry, a resultant osmotic pressure gradient appears, causing osmotic transport vertically upwards or vertically downwards. This means that the CBL/M_l_/CBL and CBL/M_r_/CBL complexes are asymmetric. Several questions therefore arise: (i) whether the asymmetry of the CBL/M_l_/CBL and CBL/M_r_/CBL complexes can be a source of electromotive force, (ii) whether the value and sign of this force depends on the concentration and composition of the solutions in the inter-membrane compartment, (iii) whether a two-membrane system constructed in this way can exhibit regulatory properties and can act as a gravireceptor in terms of free convection. In order to answer these questions, a suitable mathematical model was developed and appropriate experimental tests were carried out using measurement set-up containing aqueous solutions of NaCl or NaCl and ethanol at appropriately selected concentrations.

The purpose of this study was to develop a model of equations for the membrane potential difference (${∆\psi }_{s}$) generated in a double-membrane electrochemical cell for concentration polarization conditions based on the Kedem–Katchalsky formalism. The basic equation of this model includes the unknown solution concentration ratios at the membrane/CBLs boundaries: $C_{mr}^{B}C_{l}^{B}$($C_{r}^{B}C_{ml}^{B}$)^−1^. In this paper, we present an original procedure for calculating these concentration ratios using the transport parameters of the membrane ($L_{p}$, σ, ω), the solutions (ρ, ν), the thickness of the concentration boundary layers (δ), the concentration Rayleigh number ($R_{c}$), the concentration polarization factor ($\zeta_{s}$), the volume flux ($J_{v}$) and the ratio of known solution concentrations ($C_{m}{C_{r}}^{-1}$). We used the resulting equation to calculate the characteristics $∆\psi_{S}^{i}$ = *f*(*t*), $\psi_{S}$ = *f* (ln C_m_), $\psi_{S}$ = *f*${{(C}_{m}{C_{0}}^{-1})}_{C_{0}=const}$*,* $\psi_{S}$ = *f*${{(C}_{mn}{C_{0n}}^{-1})}_{C_{me}=const}$ and $\psi_{S}$ = *f*${{(C}_{me}{C_{0e}}^{-1})}_{C_{mn}=const}$ based on experimentally determined, in a series of independent experiments, membrane transport parameters ($L_{p}$, σ, ω) and characteristics ${∆\tau }_{m}=f(C_{m}{C_{0}}^{-1})$, $\zeta_{l}^{B}=f(C_{m}{C_{0}}^{-1})$, $\zeta_{r}^{B}=f(C_{m}{C_{0}}^{-1})$, $\zeta_{le}^{B}=f(C_{me}{C_{0}}^{-1})$ and $\zeta_{re}^{B}=f(C_{me}{C_{0}}^{-1})$. The characteristics obtained from the mathematical model were verified experimentally for aqueous solutions of NaCl and ethanol. The obtained results indicate that, due to the alteration in the configuration of the membrane system, the composition and concentration of the solutions generated a gravielectric effect and changed the sign of the membrane potential from positive to negative or vice versa.

## 2. Materials and Methods

### 2.1. Membrane System

**Figure 1:**
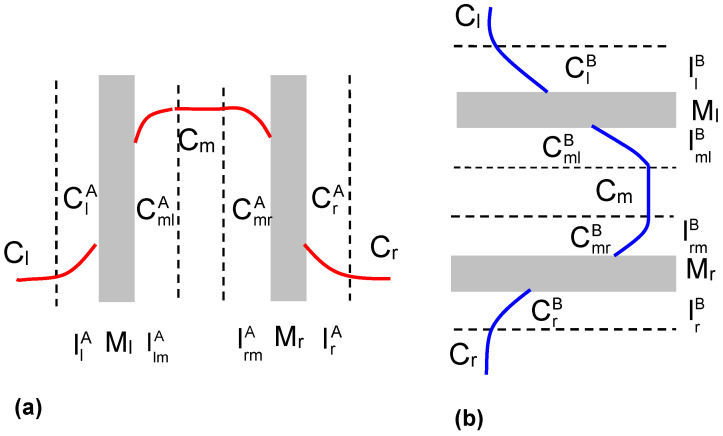
Membrane system: M_l_, M_r_—membranes; $l_{l}^{A}$, $l_{lm}^{A}$, $l_{rm}^{A}$, $l_{r}^{A}$—CBLs in configuration *A* (**a**); $l_{l}^{B}$, $l_{lm}^{B}$, $l_{rm}^{B}$, $l_{r}^{B}$—CBLs in configuration *B* (**b**); $C_{l}$, $C_{m}$, $C_{r}$—concentrations of solutions outside CBLs; $C_{l}^{A}$—concentration of solution at $l_{l}^{A}$/M_l_ boundary; $C_{ml}^{A}$—concentration of solution at M_l_/$l_{lm}^{A}$; boundary; $C_{mr}^{A}$—concentration of the solution at the border of $l_{rm}^{A}$/M_r_; $C_{r}^{A}$—concentration of the solution at the border of M_r_/$l_{r}^{A}$; $C_{l}^{B}$—concentration of the solution at the border of $l_{l}^{B}$/M_l_; $C_{ml}^{B}$—concentration of the solution at the border of M_l_/$l_{lm}^{B}$; $C_{mr}^{A}$—concentration of the solution at the border of $l_{rm}^{B}$/M_r_; $C_{r}^{B}$—concentration of the solution at the border of M_r_/$l_{r}^{B}$. Hypothetical concentration profiles are indicated by color lines in configuration *A* (red) and *B* (blue).

A double-membrane electrochemical cell in configuration *A* and *B* is illustrated schematically in Figure 1. Configuration *A* is illustrated by Figure 1a and configuration *B* is illustrated by Figure 1b. In this cell, symmetric and electrically inert membranes M_l_ and M_r_ separate three equal and sufficiently large volumes of solutions of the same electrolytic substance, in which no chemical reactions take place. At the initial moment (t = 0), the solutions are homogeneous and their concentrations are $C_{l}$ and $C_{r}$ (in the outer compartments) and $C_{m}$ (in the middle compartment). We assume that the concentrations of $C_{l}$, $C_{m}$ and $C_{r}$ satisfy the relation $C_{l}$ = $C_{r}$ = $C_{0}$ ≤ $C_{m}$. For t > 0, concentration boundary layers (CBLs) begin to form on both sides of each membrane as a result of molecular diffusion and osmosis. In configuration *A*, the process of CBLs formation is completed as soon as the cell reaches steady state and free convection processes appear. This means that the complexes $l_{l}^{A}$/M_l_/$l_{lm}^{A}$ and $l_{mr}^{A}$/M_r_/$l_{r}^{A}$ are in a state of hydrodynamic instability. Natural convection, the appearance or disappearance of which is controlled by the concentration Rayleigh number, is the cause of the partial destruction of these layers formed on both sides of the M_l_ and M_r_ membranes. The gravity vector $\vec{g}$ is parallel and tangent to the planes in which the M_l_ and M_r_ membranes are aligned and the planes in which the CBLs $l_{l}^{A}$, $l_{lm}^{A}$, $l_{rm}^{A}$ and $l_{r}^{A}$ are formed. 

Thus, for the *A* configuration (Figure 1a), the concentration at the $l_{l}^{A}$/M_l_ boundary increases from the $C_{l}$ value to the $C_{l}^{A}$, value, while the concentration at the M_l_/$l_{ml}^{A}$ boundary decreases from the $C_{m}$ value to the $C_{ml}^{A}$ value. In contrast, the concentration at the $l_{mr}^{A}$/M_r_ boundary decreases from the $C_{m}$ value to the $C_{mr}^{A}$ value, while the concentration at the M_r_/$l_{r}^{A}$ boundary increases from the $C_{m}$ value to the $C_{ml}^{A}$ value. If we assume that $C_{l}$ = $C_{r}$ = $C_{0}$, then $C_{l}^{A}$ = $C_{r}^{A}$ and $C_{ml}^{A}$ = $C_{mr}^{A}$. Thus, the complexes $l_{l}^{A}$/M_l_/$l_{lm}^{A}$ and $l_{mr}^{A}$/M_r_/$l_{r}^{A}$ are symmetrical. This means that the double-membrane electrochemical cell set in the *A* configuration is isoelectric and isoosmotic.

The process of layer formation in the *B* configuration (Figure 1b) is different. In this case, the gravity vector $\vec{g}$ is perpendicular to the planes of membranes M_l_ and M_r_ and the planes with $l_{l}^{B}$, $l_{lm}^{B}$, $l_{rm}^{B}$ and $l_{r}^{B}$ are formed. In the case of the $l_{l}^{B}$/M_l_/$l_{lm}^{B}$ complex, $C_{l}$ < $C_{l}^{B}$, $C_{ml}^{B}$ < $C_{m}$, which causes the concentration gradient vector (and therefore the density) and the gravity vector to be directed antiparallel to each other. Therefore, the $l_{l}^{B}$/M_l_/$l_{lm}^{B}$ complex is in a state of hydrodynamic stability.

In the case of the $l_{rm}^{B}$/M_l_/$l_{r}^{B}$ complex, the relations $C_{mr}^{B}$ < $C_{m}$ and $C_{r}$ < $C_{r}^{B}$ are satisfied, which results in the concentration (and therefore density) gradient vector and gravity vector being directed parallel to each other. Consequently, the $l_{rm}^{B}$/M_l_/$l_{r}^{B}$ complex is in a state of hydrodynamic instability. Again, natural convection, the appearance or disappearance of which is controlled by the $R_{c}$, is the cause of partial destruction of these layers formed on both sides of the M_l_ and M_r_ membranes. 

For the *B* configuration, the concentration at the $l_{l}^{B}$/M_l_ boundary increases from the $C_{l}$ value to the $C_{l}^{B}$ value, while the concentration at the M_l_/$l_{lm}^{B}$ boundary decreases from the $C_{m}$ value to the $C_{ml}^{B}$ value. In contrast, the concentration at the $l_{mr}^{B}$/M_r_ boundary decreases from the $C_{m}$ value to the $C_{mr}^{B}$ value, while the concentration at the M_r_/$l_{r}^{B}$ boundary increases from the $C_{m}$ value to the $C_{ml}^{B}$ value. If we assume that $C_{l}$ = $C_{r}$ = $C_{0}$, then $C_{l}^{B}$ > $C_{r}^{B}$ and $C_{ml}^{B}$ < $C_{mr}^{B}$. Thus, the complexes $l_{l}^{B}$/M_l_/$l_{lm}^{B}$ and $l_{mr}^{B}$/M_r_/$l_{r}^{B}$ are asymmetric. This means that, in a two-membrane electrochemical cell set in the *B* configuration, a gravielectric potential is generated due to the appearance or disappearance of hydrodynamic instability of one of the complexes of $l_{l}^{B}$/M_l_/$l_{lm}^{B}$ or $l_{mr}^{B}$/M_r_/$l_{r}^{B}$.

One of the most convenient tools for analyzing transport in membrane systems is the Kedem–Katchalsky formalism. For binary electrolyte solutions, the basis of this formalism is the equations describing the volume flux ($J_{v}$), solute flux ($J_{s}$) and electric charge flux ($I_{q}$). These equations are of the form [17,31]

(1) $$J_{v}=L_{p}\left[\gamma \sigma_{m}RT\left(C_{h}-C_{l}\right)+\frac{P_{E}}{\kappa_{m}}I_{m}-∆P\right]$$

(2) $$J_{s}=\omega_{m}RT\left(C_{h}-C_{l}\right)+\bar{C}\left(1-\sigma_{m}\right)J_{v}+\frac{\tau_{mj}}{z_{j}\nu_{j}F}I_{m}$$

(3) $$I_{q}=-P_{E}J_{v}+\frac{\tau_{mj}\kappa_{m}}{z_{j}\nu_{j}F}∆\mu_{m}+\kappa_{m}E$$

where $J_{v}$—volume flux; $J_{s}$—solute flux; $I_{q}$—electric charge flux; $L_{p}$, $\sigma_{m}$,$\;P_{E}$ and $\omega_{m}$—coefficients of hydraulic permeability, reflection, electroosmotic permeability and solute permeability, respectively; γ—Van’t Hoff coefficient; *RT*—the product of the gas constant and the absolute temperature; $C_{h}$ and $C_{l}$—solution concentrations ($C_{h}$ > $C_{l}$); $\kappa_{m}$—electrical conductivity; $\tau_{mj}$, $z_{mj}$, $\nu_{j}$—transfer number, valence and ion number, respectively; $\bar{C}=(C_{h}-C_{l}){\left(lnC_{h}{C_{l}}^{-1}\right)}^{-1}$ ≈ 0.5 ($C_{h}+C_{l}$)—average concentration of the solution. 

Equation (3) can be transformed to the form

(4) $$\;∆\psi_{m}=\frac{I_{m}}{\kappa_{m}}-\frac{RT}{F}{∆\tau }_{m}ln\frac{C_{h}}{C_{l}}$$

where $∆\psi_{m}$—potential difference measured with two reversible electrodes; ${∆\tau }_{m}=\tau_{ma}-\tau_{mc}$, $t_{ma}$, $\tau_{mc}$—transfer number of anion (a) and cation (c) in the membrane, respectively; $\tau_{ma}+\tau_{mc}=1$.

### 2.2. Mathematical Model of Membrane Potential

Let us consider the double-membrane electrochemical cell shown in Figure 1a,b. This cell consists of two single-membrane cells with a common compartment (m), one of which contains membrane M_l_ and the other membrane M_r_. The double-membrane electrochemical cell is filled with solutions of the same electrolytic substance of different concentrations, satisfying the condition $C_{l}$ ≤ $C_{m}$ ≤ $C_{r}$. Solutions with concentrations of $C_{l}$ and $C_{m}$ are separated by the M_l_ membrane, while solutions with concentrations of $C_{m}$ and $C_{r}$ are separated by the M_r_ membrane.

Using the procedure outlined in previous papers [16,17] and Equation (4) for the situation shown in Figure 1a, the $l_{l}^{A}$/M_l_/$l_{lm}^{A}$ complex can be written in the following forms:

(5) $$∆\psi_{l}^{A}=\frac{I_{l}^{A}}{\kappa_{l}^{A}}-\frac{RT}{F}\left(\tau_{la}-\tau_{lc}\right)ln\frac{C_{l}^{A}}{C_{l}}$$

(6) $$∆\psi_{Ml}^{A}=\frac{I_{Ml}^{A}}{\kappa_{Ml}^{A}}-\frac{RT}{F}\left(\tau_{Mla}-\tau_{Mlc}\right)ln\frac{C_{ml}^{A}}{C_{l}^{A}}$$

(7) $$∆\psi_{lm}^{A}=\frac{I_{lm}^{A}}{\kappa_{lm}^{A}}-\frac{RT}{F}\left(\tau_{lma}-\tau_{lmc}\right)ln\frac{C_{m}^{A}}{C_{ml}^{A}}$$

where $∆\psi_{l}^{A}$—electrical potential difference across the $l_{l}^{A}$ layer; $∆\psi_{Ml}^{A}$—electrical potential difference across the M_l_ membrane; $∆\psi_{lm}^{A}$—electrical potential difference across the $l_{lm}^{A}$ layer; $I_{l}^{A}$—ionic current through layer $l_{l}^{A}$; $I_{Ml}^{A}$—ionic current through membrane M_l_; $I_{lm}^{A}$—ionic current through layer $l_{lm}^{A}$; $\kappa_{l}^{A}$—electrical conductivity coefficient of layer $l_{l}^{A}$; $\kappa_{Ml}^{A}$—electrical conductivity coefficient of membrane M_l_; $\kappa_{lm}^{A}$—electrical conductivity coefficient of layer $l_{lm}^{A}$; $\tau_{la}$, $\tau_{lc}$, $\tau_{lma}$, $\tau_{lmc}$—transfer numbers of anions (a) and cations (c) in layers $l_{l}^{A}$ and $l_{lm}^{A}$; $\tau_{Mla}$, $\tau_{Mlc}$—transfer numbers of anions (a) and cations (c) in the M_l_ membrane; *RT*—product of gas constant and absolute temperature; *F*—Faraday’s constant.

In the steady state, the following conditions are fulfilled:

(8) $$\Delta \psi_{\mathrm{Sl}}^{A}=\Delta \psi_{l}^{A}+\Delta \psi_{\mathrm{Ml}}^{A}+\Delta \psi_{\mathrm{lm}}^{A}$$

(9) $$I_{l}^{A}=I_{\mathrm{Ml}}^{A}=I_{\mathrm{lm}}^{A}=I_{\mathrm{Sl}}^{A}=\mathrm{const}$$

Based on Equations (5)–(7) and the conditions $\tau_{l}$ = $\tau_{lm}$ = $\tau_{0}$, we obtain

(10) $$∆\psi_{Sl}^{A}=\frac{I_{Sl}^{A}}{\kappa_{Sl}^{A}}-\frac{RT}{F}\left[{∆\tau }_{0}ln\frac{C_{m}}{C_{l}}+\left({∆\tau }_{Ml}-{∆\tau }_{0}\right)ln\frac{C_{ml}^{A}}{C_{l}^{A}}\right]$$

where ${∆\tau }_{0}=\tau_{0a}-\tau_{0c}$, ${∆\tau }_{Ml}=\tau_{ma}-\tau_{mc}$, $\kappa_{Sl}^{A}=\kappa_{l}^{A}\kappa_{Ml}^{A}\kappa_{ml}^{A}{\left(\kappa_{Ml}^{A}\kappa_{ml}^{A}+\kappa_{l}^{A}\kappa_{ml}^{A}+\kappa_{l}^{A}\kappa_{Ml}^{A}\right)}^{-1}$. 

To obtain the equation for the $l_{mr}^{A}$/M_r_/$l_{r}^{A}$ complex, it is necessary to replace the subscript l in Equations (5)–(7) with the subscript *r* and repeat the procedure illustrated by Equations (5)–(10). The result of such an operation is the equation

(11) $$∆\psi_{Sr}^{A}=\frac{I_{Sr}^{A}}{\kappa_{Sr}^{A}}-\frac{RT}{F}\left[{∆\tau }_{0}ln\frac{C_{m}}{C_{r}}+\left({∆\tau }_{Mr}-{∆\tau }_{0}\right)ln\frac{C_{mr}^{A}}{C_{r}^{A}}\right]$$

where $\tau_{r}$ = $\tau_{rm}$ = $\tau_{0}$, ${∆\tau }_{0}=\tau_{0a}-\tau_{0c}$, ${∆\tau }_{Mr}=\tau_{ma}-\tau_{mc}$, $\kappa_{Sr}^{A}=\kappa_{r}^{A}\kappa_{Mr}^{A}\kappa_{mr}^{A}{\left(\kappa_{Mr}^{A}\kappa_{mr}^{A}+\kappa_{r}^{A}\kappa_{mr}^{A}+\kappa_{r}^{A}\kappa_{Mr}^{A}\right)}^{-1}$.

Subtracting Equations (10) and (11) with sides and assuming that ${∆\tau }_{Mr}$ = ${∆\tau }_{Ml}$ = ${∆\tau }_{M}$, we obtain the following:

(12) $$∆\psi_{S}^{A}=∆\psi_{Sl}^{A}-∆\psi_{Sr}^{A}=\frac{I_{Sl}^{A}}{\kappa_{Sl}^{A}}-\frac{I_{Sr}^{A}}{\kappa_{Sr}^{A}}+\frac{RT}{F}\left[{∆\tau }_{0}ln\frac{C_{l}}{C_{r}}+\left({∆\tau }_{M}-{∆\tau }_{0}\right)ln\frac{C_{mr}^{A}C_{l}^{A}}{C_{r}^{A}C_{ml}^{A}}\right]$$

The above equation describes the difference in electrical potentials generated in a system of two membranes aligned in horizontal planes perpendicular to the gravity vector.

For the situation shown in Figure 1b, Equation (12) takes the following form:

(13) $$∆\psi_{S}^{B}=∆\psi_{Sl}^{B}-∆\psi_{Sr}^{B}=\frac{I_{Sl}^{B}}{\kappa_{Sl}^{B}}-\frac{I_{Sr}^{B}}{\kappa_{Sr}^{B}}+\frac{RT}{F}\left[{∆\tau }_{0}ln\frac{C_{l}}{C_{r}}+\left({∆\tau }_{M}-{∆\tau }_{0}\right)ln\frac{C_{mr}^{B}C_{l}^{B}}{C_{r}^{B}C_{ml}^{B}}\right]$$

Thus, the change in membrane potential when the double-membrane electrochemical cell is reoriented from the *A* to *B* configuration is

(14) $$∆\psi_{S}=∆\psi_{S}^{B}-∆\psi_{S}^{A}=\frac{I_{Sl}^{B}}{\kappa_{Sl}^{B}}-\frac{I_{Sr}^{B}}{\kappa_{Sr}^{B}}-\frac{I_{Sl}^{A}}{\kappa_{Sl}^{A}}+\frac{I_{Sr}^{A}}{\kappa_{Sr}^{A}}+\frac{RT}{F}\left(∆\tau_{m}-∆\tau_{0}\right)ln\left(\frac{C_{mr}^{B}C_{l}^{B}}{C_{r}^{B}C_{ml}^{B}}\frac{C_{r}^{A}C_{ml}^{A}}{C_{mr}^{A}C_{l}^{A}}\right)$$

Consider the case where the electrochemical cell contains two equal membranes and that the outer compartments (l, r) contain solutions whose concentrations satisfy the condition $C_{l}$ = $C_{r}$ = $C_{0}$. In configuration *A*, the complexes $l_{l}^{A}$/M_l_/$l_{lm}^{A}$ and $l_{mr}^{A}$/M_r_/$l_{r}^{A}$ are symmetrical. This means that $I_{Sl}^{A}=I_{Sr}^{A}=I_{S}^{A}$ and $\kappa_{Sr}^{A}$ = $\kappa_{Sl}^{A}$, $C_{mr}^{A}$ = $C_{ml}^{A}$ and $C_{l}^{A}$ = $C_{r}^{A}$. Considering these conditions in Equation (12), we obtain $∆\psi_{S}^{A}=0$.

In the *B* configuration, on the other hand, the $l_{l}^{B}$/M_l_/$l_{lm}^{B}$ and $l_{mr}^{B}$/M_r_/$l_{r}^{B}$ complexes are asymmetric. This means that $C_{mr}^{B}$ > $C_{ml}^{B}$ and $C_{r}^{B}$ > $C_{l}^{B}$ but $I_{Sl}^{B}=I_{Sr}^{B}=I_{S}^{B}$. Thus, $∆\psi_{S}^{B}$ ≠ 0. Given the above conditions in Equation (14), we obtain

(15) $$∆\psi_{S}=∆\psi_{S}^{B}=\frac{RT}{F}\left(∆\tau_{M}-∆\tau_{0}\right)ln\frac{C_{mr}^{B}C_{l}^{B}}{C_{r}^{B}C_{ml}^{B}}$$

Based on the classical and modified [14] forms of Equation (2) and the amperostatic condition ($I_{l}$ = $I_{m}$= $I_{r}$ = 0), we can write

(16) $$J_{l}^{B}=\frac{D_{l}}{\delta_{l}^{B}}\left(C_{l}^{B}-C_{l}\right)+\frac{1}{2}\left(C_{l}^{B}+C_{l}\right)J_{vl}^{B}$$

(17) $$J_{sl}^{B}=\omega RT\zeta_{l}^{B}\left(C_{m}-C_{l}\right)+\frac{1}{2}\left(C_{m}+C_{l}^{B}\right)J_{vsl}^{B}$$

(18) $$J_{ml}^{B}=\frac{D_{m}}{\delta_{ml}^{B}}\left(C_{m}{-C}_{ml}^{B}\right)+\frac{1}{2}\left({C_{m}+C}_{ml}^{B}\right)J_{vml}^{B}$$

where $J_{l}^{B}$—soute flux through $l_{l}^{B}$ layer, $J_{sl}^{B}$—solute flux through $l_{l}^{B}$/M_l_/$l_{lm}^{B}$ complex, $J_{ml}^{B}$—solute flux through $l_{lm}^{B}$ layer, $J_{vl}^{B}$—volume flux through $l_{l}^{B}$ layer, $J_{vsl}^{B}$—volume flux through $l_{l}^{B}$/M_l_/$l_{lm}^{B}$ complex, $J_{vml}^{B}$—volume flux through $l_{lm}^{B}$ layer.

In order to obtain a set of equations for the complex $l_{mr}^{A}$/M_r_/$l_{r}^{A}$, it is necessary to replace the subscript *l* in Equations (5)–(9) with the subscript *r*. As a result of such an operation, we obtain the following:

(19) $$J_{rm}^{B}=\frac{D_{rm}}{\delta_{rm}^{B}}\left(C_{m}{-C}_{mr}^{B}\right)+\frac{1}{2}\left({C_{m}+C}_{mr}^{B}\right)J_{vmr}^{B}$$

(20) $$J_{sr}^{B}=\omega RT\zeta_{r}^{B}\left(C_{m}-C_{r}\right)+\frac{1}{2}\left(C_{m}+C_{r}^{B}\right)J_{vsr}^{B}$$

(21) $$J_{r}^{B}=\frac{D_{r}}{\delta_{r}^{B}}\left(C_{r}^{B}-C_{r}\right)+\frac{1}{2}\left(C_{r}^{B}+C_{r}\right)J_{vr}^{B}$$

In steady state, the conditions are fulfilled by

(22) $$J_{l}^{B}=J_{sl}^{B}=J_{ml}^{B}$$

(23) $$J_{r}^{B}=J_{sr}^{B}=J_{mr}^{B}$$

(24) $$J_{vl}^{B}=J_{vs}^{B}=J_{vml}^{B}=J_{vr}^{B}=J_{vs}^{B}=J_{vmr}^{B}=J_{v}$$

Considering Equations (16)–(18) in Equation (22) and Equations (19)–(21) in Equation (23), we obtain the following expression:

(25) $$\frac{C_{l}^{B}}{C_{ml}^{B}}=\frac{C_{l}}{C_{m}}\left(\frac{\alpha_{0}+\alpha_{1}J_{v}+\alpha_{2}{J_{v}}^{2}}{\beta_{0}+\beta_{1}J_{v}+\beta_{2}{J_{v}}^{2}}\right)$$

where

$\alpha_{0}=\frac{D_{ml}}{\delta_{ml}^{B}}\left[\frac{D_{l}}{\delta_{l}^{B}}+\zeta_{l}^{B}\omega RT\left(\frac{C_{m}}{C_{l}}-1\right)\right]$, $\alpha_{1}=-\frac{1}{2}\left\{\frac{D_{l}}{\delta_{l}^{B}}+\zeta_{l}^{B}\omega RT\left(\frac{C_{m}}{C_{l}}-1\right)-\left[\frac{C_{m}}{C_{l}}\left(1-\sigma \right)-\sigma \right]\frac{D_{ml}}{\delta_{ml}^{B}}\right\}$, 

$\alpha_{2}=-\frac{1}{4}\left[\frac{C_{m}}{C_{l}}\left(1-\sigma \right)-\sigma \right]$, $\beta_{0}=\frac{D_{l}}{\delta_{l}^{B}}\left[\frac{D_{ml}}{\delta_{ml}^{B}}-\zeta_{l}^{B}\omega RT\left(1-\frac{C_{l}}{C_{m}}\right)\right]$, 

$\beta_{1}=-\frac{1}{2}\left\{\zeta_{l}^{B}\omega RT\left(1-\frac{C_{l}}{C_{m}}\right)-\frac{D_{ml}}{\delta_{ml}^{B}}+\left[\frac{C_{l}}{C_{m}}\left(1-\sigma \right)-\sigma \right]\frac{D_{l}}{\delta_{l}^{B}}\right\}$, $\beta_{2}=-\frac{1}{4}\left[\frac{C_{l}}{C_{m}}\left(1-\sigma \right)-\sigma \right]$

(26) $$\frac{C_{mr}^{B}}{C_{r}^{B}}=\frac{C_{m}}{C_{r}}\left(\frac{\gamma_{0}+\gamma_{1}J_{v}+\gamma_{2}{J_{v}}^{2}}{\varepsilon_{0}+\varepsilon_{1}J_{v}+\varepsilon_{2}{J_{v}}^{2}}\right)$$

where

$\gamma_{0}=\frac{D_{r}}{\delta_{r}^{B}}\left[\frac{D_{mr}}{\delta_{mr}^{B}}-\zeta_{r}^{B}\omega RT\left(1-\frac{C_{r}}{C_{m}}\right)\right]$, $\gamma_{1}=-\frac{1}{2}\left\{\zeta_{r}^{B}\omega RT\left(1-\frac{C_{r}}{C_{m}}\right)-\frac{D_{mr}}{\delta_{mr}^{B}}+\left[\frac{C_{r}}{C_{m}}\left(1-\sigma \right)-\sigma \right]\frac{D_{r}}{\delta_{r}^{B}}\right\}$, 

$\gamma_{2}=-\frac{1}{4}\left[\frac{C_{r}}{C_{m}}\left(1-\sigma \right)-\sigma \right]$, $\varepsilon_{0}=\frac{D_{mr}}{\delta_{mr}^{B}}\left[\frac{D_{r}}{\delta_{r}^{B}}+\zeta_{l}^{B}\omega RT\left(\frac{C_{m}}{C_{r}}-1\right)\right]$, 

$\varepsilon_{1}=-\frac{1}{2}\left\{\zeta_{l}^{B}\omega RT\left(\frac{C_{m}}{C_{r}}-1\right)+\frac{D_{r}}{\delta_{r}^{B}}-\left[\frac{C_{m}}{C_{r}}\left(1-\sigma \right)-\sigma \right]\frac{D_{mr}}{\delta_{mr}^{B}}\right\}$, $\varepsilon_{2}=-\frac{1}{4}\left[\frac{C_{m}}{C_{r}}\left(1-\sigma \right)-\sigma \right]$.

Multiplying Equations (25) and (26) by sides, we obtain the following expression:

(27) $$\frac{C_{l}^{B}}{C_{ml}^{B}}\frac{C_{mr}^{B}}{C_{r}^{B}}=\frac{C_{l}}{C_{r}}\left(\frac{\alpha_{0}+\alpha_{1}J_{v}+\alpha_{2}{J_{v}}^{2}}{\beta_{0}+\beta_{1}J_{v}+\beta_{2}{J_{v}}^{2}}\right)\left(\frac{\gamma_{0}+\gamma_{1}J_{v}+\gamma_{2}{J_{v}}^{2}}{\varepsilon_{0}+\varepsilon_{1}J_{v}+\varepsilon_{2}{J_{v}}^{2}}\right)$$

For conditions $J_{v}$ = 0 and $\delta_{l}^{B}=D_{l}\left(1-\zeta_{l}^{B}\right){\left(2RT{\omega \zeta }_{l}^{B}\right)}^{-1}$, $\delta_{lm}^{B}=D_{lm}\left(1-\zeta_{l}^{B}\right){\left(2RT{\omega \zeta }_{l}^{B}\right)}^{-1}$, $\delta_{r}^{B}=D_{r}\left(1-\zeta_{r}^{B}\right){\left(2RT{\omega \zeta }_{r}^{B}\right)}^{-1}$ and $\delta_{rm}^{B}=D_{rm}\left(1-\zeta_{r}^{B}\right){\left(2RT{\omega \zeta }_{r}^{B}\right)}^{-1}$, Equation (27) is simplified to form

(28) $$\frac{C_{mr}^{B}}{C_{r}^{B}}\frac{C_{l}^{B}}{C_{ml}^{B}}=\frac{C_{l}}{C_{r}}\left[\frac{1-\frac{1}{2}\left(1-\zeta_{r}^{B}\right)\left(1-\frac{C_{r}\;}{C_{m}}\right)}{1+\frac{1}{2}\left(1-\zeta_{r}^{B}\right)\left(\frac{C_{m}\;}{C_{r}}-1\right)}\right]\left[\frac{1+\frac{1}{2}\left(1-\zeta_{l}^{B}\right)\left(\frac{C_{m}\;}{C_{l}}-1\right)}{1-\frac{1}{2}\left(1-\zeta_{l}^{B}\right)\left(1-\frac{C_{l}\;}{C_{m}}\right)}\right]$$

Suppose that, for dilute solutions, $J_{vl}^{B}$ = $J_{vs}^{B}$ = $J_{vml}^{B}$ = 0. Then, based on Equations (25) and (26), we obtain

(29) $$\frac{C_{l}^{B}}{C_{ml}^{B}}=\frac{C_{l}}{C_{m}}\left[\frac{1+\frac{\delta_{l}^{B}}{D_{l}}\omega RT\zeta_{l}^{B}\left(\frac{C_{m}}{C_{l}}-1\right)}{1-\frac{\delta_{ml}^{B}}{D_{ml}}\omega RT\zeta_{l}^{B}\left(1-\frac{C_{l}}{C_{m}}\right)}\right]$$

(30) $$\frac{C_{mr}^{B}}{C_{r}^{B}}=\frac{C_{m}}{C_{r}}\left[\frac{1-\frac{\delta_{mr}^{B}}{D_{mr}}\omega RT\zeta_{r}^{B}\left(1-\frac{C_{r}}{C_{m}}\right)}{1+\frac{\delta_{r}^{B}}{D_{r}}\omega RT\zeta_{r}^{B}\left(\frac{C_{m}}{C_{r}}-1\right)}\right]$$

Given the conditions $\delta_{ml}^{B}$ = $\delta_{l}^{B},\delta_{mr}^{B}$ = $\delta_{r}^{B}$, $D_{l}$ = $D_{ml}$ = $D_{mr}$ = $D_{r}$ = D in Equations (27) and (28) and the expressions $\delta_{l}^{B}=D_{l}\left(1-\zeta_{l}^{B}\right){\left(2RT{\omega \zeta }_{l}^{B}\right)}^{-1}$, $\delta_{lm}^{B}=D_{lm}\left(1-\zeta_{l}^{B}\right){\left(2RT{\omega \zeta }_{l}^{B}\right)}^{-1}$, $\delta_{r}^{B}=D_{r}\left(1-\zeta_{r}^{B}\right){\left(2RT{\omega \zeta }_{r}^{B}\right)}^{-1}$, $\delta_{rm}^{B}=D_{rm}\left(1-\zeta_{r}^{B}\right){\left(2RT{\omega \zeta }_{r}^{B}\right)}^{-1}$ we obtain

(31) $$\frac{C_{mr}^{B}}{C_{r}^{B}}\frac{C_{l}^{B}}{C_{ml}^{B}}=\frac{C_{l}}{C_{r}}\left[\frac{1-\frac{1}{2}\left(1-\zeta_{r}^{B}\right)\left(1-\frac{C_{r}\;}{C_{m}}\right)}{1+\frac{1}{2}\left(1-\zeta_{r}^{B}\right)\left(\frac{C_{m}\;}{C_{r}}-1\right)}\right]\left[\frac{1+\frac{1}{2}\left(1-\zeta_{l}^{B}\right)\left(\frac{C_{m}\;}{C_{l}}-1\right)}{1-\frac{1}{2}\left(1-\zeta_{l}^{B}\right)\left(1-\frac{C_{l}\;}{C_{m}}\right)}\right]$$

The coefficients $\zeta_{l}^{B}$ and $\zeta_{r}^{B}$ can also be calculated from the expressions in [17].

(32) $$\zeta_{l}^{B}=C_{l}\left[g{D_{l}}^{2}\frac{\partial \rho }{\partial C}\left(\frac{C_{m}}{C_{l}}-1\right)\right]{\left[16R_{Cl}{\left(RT\right)}^{2}\omega^{3}\rho_{l}\nu_{l}\right]}^{-\frac{1}{3}}$$

(33) $$\zeta_{r}^{B}=C_{m}\left[g{D_{r}}^{2}\frac{\partial \rho }{\partial C}\left(1-\frac{C_{r}}{C_{m}}\right)\right]{\left[16R_{Cr}{\left(RT\right)}^{2}\omega^{3}\rho_{r}\nu_{r}\right]}^{-\frac{1}{3}}$$

### 2.3. Measurement System

**Figure 2:**
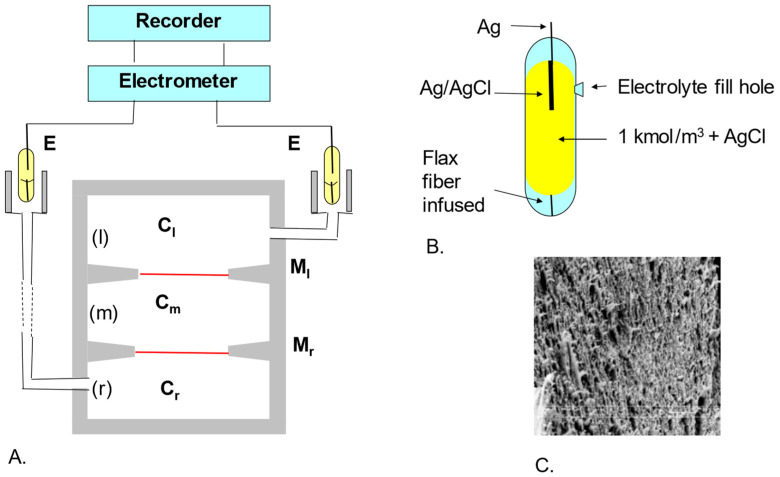
Measurement set. (**A**) Ml, Mr—membranes; E—electrodes, (l), (m), (r)—measuring vessels, $C_{l}$, $C_{m}$, $C_{r}$—solution concentrations; (**B**) Scheme of an electrode vessel containing an Ag/AgCl electrode immersed in a concentrated KCl solution saturated with AgCl; (**C**) Image of the Ultra Flo 145 Dialyzer membrane obtained from a scanning microscope at 10,000× magnification.

Membrane potential studies in a double-membrane physicochemical cell for concentration polarization conditions were performed using the measurement set-up shown in Figure 2. The electrochemical cell consisted of three cylindrical vessels (l), (m) and (r) of 300 cm^3^ each made of Plexiglas (Figure 2A). Vessels (l) and (r) in all experiments contained aqueous solutions of NaCl or solutions of NaCl in aqueous ethanol solution of equal concentrations. Vessel (m) was filled with an aqueous NaCl solution or NaCl solution in an aqueous ethanol solution with different concentrations of $C_{m}$. Vessels (l), (m) and (r) were separated by Ultra Flo 145 Dialyzer hemodialysis membranes (Artificial Organs Division, Travenol Laboratories S.A., Brussels, Belgium). An image taken with a Zeiss Supra 35 with magnification scanning microscope is shown in Figure 2C. The Ag/AgCl measuring electrodes were placed in a glass vessel filled with a 1 kmol m^−3^ aqueous KCl solution saturated with AgCl (Figure 2B). The contact between the E electrodes and the $C_{l}$ and $C_{r}$ solutions was made via a concentrated KCl solution saturated with AgCl and flax fiber. In all experiments, the electrodes (E) were positioned vertically. The electrochemical cell and electrodes were placed in a thermostated electrostatic shield made of copper sheet. The shield was grounded. Measurements of electrical potentials were carried out under isothermal (*T* = const = 295 K) and iso-osmotic ($J_{v}$ = 0) conditions using an electrometer, and the results were recorded using a recorder to which a computer was connected. 

The measurement procedure was divided into three stages. In the first stage, the electrochemical cell was set up so that the membranes were oriented in vertical planes. We denoted this configuration by *A* (see Figure 1a). After obtaining a steady state, the electrochemical cell was set up so that the membranes were oriented in horizontal planes (second stage). We denoted this configuration by *B* (see Figure 1b). After obtaining a steady state, the electrochemical cell was repositioned so that the membranes were oriented in vertical planes (configuration *A*). In all steps, the orientation of the measuring electrodes was not changed. In each of these configurations, the potentials $∆\psi_{S}^{B}$ and $∆\psi_{S}^{A}$ were measured and calculated $∆\psi_{S}=∆\psi_{S}^{B}-∆\psi_{S}^{A}$, which is a measure of the potential generated in a double-membrane electrochemical cell.

## 3. Results and Discussion

### 3.1. Time Dependence of Membrane Potential

**Figure 3:**
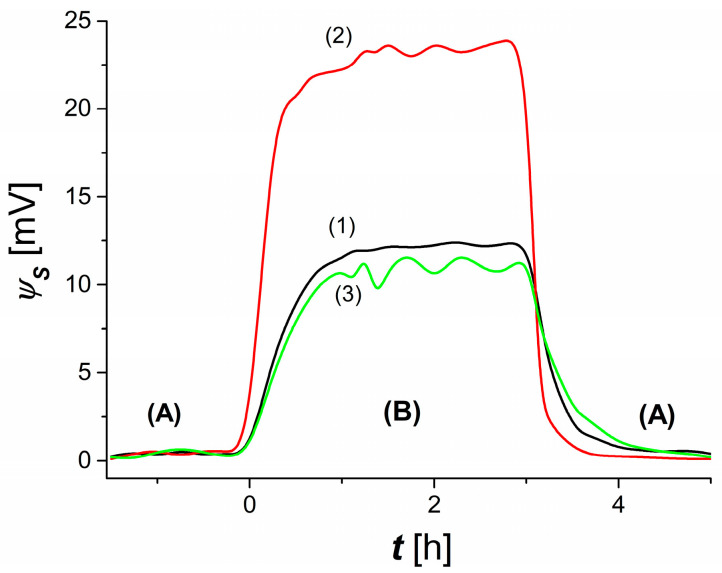
Dependences $∆\psi_{S}^{i}$ = f(t), i = A, B for configuration A (part A) and configuration B (part B). Curve 1 was obtained for $C_{m}$ = 0.5 mol m^−3^ and $C_{0}$ = 0.1 mol m^−3^, curve 2—for $C_{m}$ = 3 mol m^−3^ and $C_{0}$ = 0.1 mol m^−3^, and curve 3—for $C_{m}$ = 20 mol m^−3^ and $C_{0}$ = 1 mol m^−3^.

To show how changing the configuration of the membrane system affects the creation of the membrane potential, measurements of this potential were made successively in configuration *A*, then in configuration *B* and again in configuration *A*. In the first step, the membrane system was set in configuration *A*. After obtaining a steady state in which the potential reached $∆\psi_{S}^{A}$, the membrane system was set up in configuration *B* and the evolution of the potential was monitored until a second steady state was obtained in which the membrane potential reached $∆\psi_{S}^{B}$. In the next step, the membrane system was again set in configuration *A* and the evolution of the membrane potential was monitored until a steady state was reached. Typical characteristics of $∆\psi_{S}^{i}$ = *f*(*t*), *i* = *A, B* are shown in Figure 3. From the course of these characteristics, it can be seen that t = 0, $∆\psi_{S}^{i}$ = 0. This means that the $l_{l}^{A}$/M_l_/$l_{lm}^{A}$ and $l_{mr}^{A}$/M_r_/$l_{r}^{A}$ complexes are symmetric.

Therefore, it can be assumed that the thickness of the $l_{l}^{A}$, $l_{lm}^{A}$, $l_{rm}^{A}$ and $l_{r}^{A}$ layers is approximately equal, which can be written in the form of $\delta_{l}^{A}$, $\delta_{lm}^{A}$, $\delta_{rm}^{A}$ and $\delta_{r}^{A}$. For t > 0, there is an asymmetrization of the $l_{l}^{B}$/M_l_/$l_{lm}^{B}$ and $l_{mr}^{B}$/M_r_/$l_{r}^{B}$ complexes, which leads to a situation wherein the thicknesses of ${\;\delta }_{l}^{B}$, $\delta_{lm}^{B}$, $\delta_{rm}^{B}$ and $\delta_{r}^{B}$ satisfy the conditions $\delta_{l}^{B}$ > $\delta_{l}^{A}$, $\delta_{lm}^{B}$ > $\delta_{lm}^{A}$ and $\delta_{rm}^{B}$ ≈ $\delta_{rm}^{A}$ and $\delta_{r}^{B}$ ≈ $\delta_{r}^{A}$. The effect is to generate a membrane potential satisfying the condition $∆\psi_{S}^{B}$ > 0. This potential is the result of eliminating natural convection in the M_l_ membrane surroundings. Curves 1 and 2 shown in this figure show that an increase in the concentration value from $C_{m}$ = 0.5 mol m^−3^ to $C_{m}$= 3 mol m^−3^, with $C_{0}$ fixed, results in a two-fold increase in $∆\psi_{S}^{B}$ = $\psi_{S}$. In addition, for t ≥ 0.5 h, occasional fluctuations of $∆\psi_{S}^{B}$ appear. In the steady state (curves 1 and 2), natural convection occurs only in the vicinity of the membrane M_r_. From the course of curve 3, it can be seen that the simultaneous increase in the values of the concentrations $C_{m}$ and $C_{0}$ to the values of $C_{m}$ = 20 mol m^−3^ and $C_{0}$ = 1 mol m^−3^, respectively, causes a decrease in the value of $∆\psi_{S}^{B}$ and the appearance of fluctuations $∆\psi_{S}^{B}$. In this case, there is a partial elimination of natural convection in the surroundings of the M_l_ membrane.

### 3.2. Concentration Dependence of Membrane Potential

**Figure 4:**
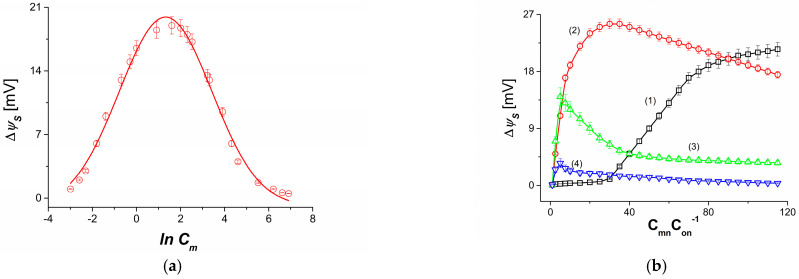
(**a**) Dependence $\psi_{S}$ = f (ln $C_{m}$) for aqueous NaCl solutions. (**b**) Dependence $\psi_{S}$ = f${{(C}_{m}{C_{0}}^{-1})}_{C_{0}=const}$ for: $C_{0}$ = 0.01 mol m^−3^ (curve 1), $C_{0}$ = 0.1 mol m^−3^ (curve 2), $C_{0}$ = 1 mol m^−3^ (curve 3) and $C_{0}$ = 10 mol m^−3^ (curve 4).

In the first of the two measurement series, the dependence $C_{m}C_{0}$^−1^ = 10 was satisfied between the concentrations of $C_{m}$ and $C_{0}$. The values of the concentrations of $C_{m}$ expressed in mol m^−3^ were as follows: 0.05; 0.5; 1; 5; 10; 50; 100; 500; 1000. In turn, the values of $C_{0}$ were as follows: 0.005; 0.01; 0.05; 0.1; 0.5; 1; 5; 10; 50; 100. Figure 4 shows the relationship $\psi_{S}$ = *f* (ln $C_{m}$). A graphical illustration of this relationship is the logarithmic curve resulting from fitting the measurement results to a Gaussian curve. The curve shown in Figure 4 shows that there are threshold (minimum and maximum) values of $C_{m}$ and therefore $C_{0}$ for which a non-zero (additive) potential $\psi_{S}$ is generated. The minimum values of $C_{m}$ and $C_{0}$ are $C_{m}^{min}$ ≈ 0.05 mol m^−3^ (ln 0.05 = −2.996), $C_{0}^{min}$ ≈ 0.005 mol m^−3^ (ln 0.005 = −5.298), $C_{m}^{min}$ ≈ 1000 mol m^−3^ (ln 1000 = 6.908) oraz $C_{0}^{min}$ ≈ 100 mol m^−3^ (ln 100 = 4.605). In contrast, the maximum values of $C_{m}$ and $C_{0}$ are $C_{m}^{max}$ = 5 mol m^−3^ (ln 5 = 1.609) and $C_{0}^{max}$ = 5 mol m^−3^ (ln 0.5 = −0.693).

Figure 4b shows the dependence $\psi_{S}$ = *f*${{(C}_{m}{C_{0}}^{-1})}_{C_{0}=const}$, with $C_{m}{C_{0}}^{-1}$ taking values from 1 to 115, while $C_{0}$ took fixed values. Curve 1 was obtained for $C_{0}$= 0.01 mol m^−3^, curve 2—for $C_{0}$ = 0.1 mol m^−3^, curve 3—for $C_{0}$= 1 mol m^−3^ and curve 4—for $C_{0}$ = 10 mol m^−3^. From the course of curve 1, it can be seen that there is an interval of $C_{m}{C_{0}}^{-1}$ in which, despite an increase in $C_{m}{C_{0}}^{-1}$, $\psi_{S}$ does not change. It is only when the threshold concentration of $C_{m}$ equal to $C_{m}^{min}$ = 0.3 mol m^−3^ is exceeded that a non-zero value of $\psi_{S}$ appears. This means that, for $C_{m}$ ≥ $C_{m}^{min}$, there is a modification of the concentration field in the M_l_ membrane cavity due to the disappearance of natural convection. In the case of curve 2, a non-zero value of $\psi_{S}$ already appears for $C_{m}{C_{0}}^{-1}$ = 1. Then, for $C_{m}{C_{0}}^{-1}$ > 1, $\psi_{S}$ increases nonlinearly until it reaches a maximum value of $\psi_{S}$ = 25 mV (for $C_{m}{C_{0}}^{-1}$ = 30). After it is exceeded, $\psi_{S}$ decreases nonlinearly. Similar to curve 2, for curves 3 and 4, a non-zero value of $\psi_{S}$ appears for $C_{m}{C_{0}}^{-1}$= 1. For $C_{m}{C_{0}}^{-1}$ > 1, $\psi_{S}$ increases nonlinearly until it reaches a maximum value of $\psi_{S}$ = 13 mV (for curve 3) and $\psi_{S}$= 3 mV (for curve 4). The values of $C_{m}{C_{0}}^{-1}$ for which curves 3 and 4 reach the maximum values of $\psi_{S}$ are $C_{m}{C_{0}}^{-1}$ = 5 and $C_{m}{C_{0}}^{-1}$= 2.5, respectively. From the course of curves 1, 2, 3 and 4, it is clear that the maxima of these curves move in the direction of decreasing $C_{m}{C_{0}}^{-1}$.

**Figure 5:**
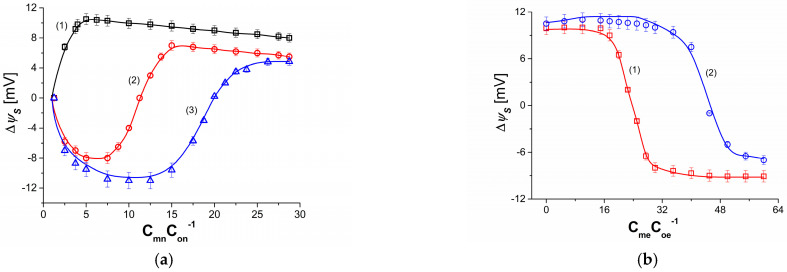
(**a**) Experimental and calculated $\psi_{S}$ = f${{(C}_{mn}{C_{0n}}^{-1})}_{C_{me}=const}$ for NaCl solutions in aqueous ethanol solution. Curve 1 was obtained for $C_{me}$ = 0, curve 2—for $C_{me}$ = 50 mol m^−3^ while curve 3—for $C_{me}$ = 100 mol m^−3^. (**b**) Experimental and calculated $\psi_{S}$ = f${{(C}_{me}{C_{0e}}^{-1})}_{C_{mn}=const}$ for ethanol solutions in aqueous NaCl solution. Curve 1 was obtained for $C_{mn}$= 5 mol m^−3^, while curve 2 was obtained for $C_{mn}$= 10 mol m^−3^. The curves are within 7% error range.

Figure 5a shows the dependences $\psi_{S}$ = *f*${{(C}_{mn}{C_{0n}}^{-1})}_{C_{me}=const}$ for NaCl solutions in an aqueous ethanol solution. Experimental results are shown with symbols (**□**, **○**, **△**). The solid lines illustrate the results calculated from Equations (15) and (28). In all experiments, $C_{0n}$ = 1 mol m^−3^, while $C_{mn}$ varied from 1 to 30 mol m^−3^. Curve 1 was obtained for $C_{me}$ = 0, curve 2—for $C_{me}$ = 50 mol m^−3^, while curve 3—for 100 mol m^−3^. Similar to the curves shown in Figure 5, curve 1 illustrating the nonlinear dependence $\psi_{S}$ = *f*${{(C}_{mn}{C_{0n}}^{-1})}_{C_{me}=const}$ includes the addition of $\psi_{S}$ values. Unlike curve 1, curves 2 and 3 contain positive and negative values of $\psi_{S}$. Curve 2 shows that, for $C_{mn}$= 11.25 mol m^−3^, $\psi_{S}\;$= 0, for $C_{mn}$< 11.25 mol m^−3^, $\psi_{S}$< 0, while for $C_{mn}$ > 11.25 mol m^−3^, $\psi_{S}$ > 0. Curve 3, on the other hand, shows that, for $C_{mn}$ = 22.3 mol m^−3^, $\psi_{S}$ = 0, for $C_{mn}$ < 22. 3 mol m^−3^, $\psi_{S}$ < 0, while for $C_{mn}$> 22.3 mol m^−3^, $\psi_{S}$ > 0. The change in the sign of $\psi_{S}$ from negative to positive is related to the switch of natural convection elimination from the $l_{mr}^{B}$/M_r_/$l_{r}^{B}$ complex to the $l_{l}^{B}$/M_l_/$l_{lm}^{B}$ complex. The functioning of this switch is based on the dependence of the density gradients of the solution located in compartment (m) to the density gradients of the solutions in compartments (l) and (r) and the gravity vector. If the density of the solution consisting of 11.25 mol m^−3^ NaCl, 50 mol m^−3^ ethanol and water, with which the compartment (m) of the measurement system is filled, is less than the density of water with which the compartments (l) and (r) are filled, then $\psi_{S}$ = 0. If, in turn, the compartment (m) contains less than 11. 25 mol m^−3^ NaCl and the same amount of ethanol, then the density of the solution is less than the density of water-filling compartments (l) and (r) and $\psi_{S}$ < 0. The membrane potential satisfies the condition $\psi_{S}$ > 0 when compartment (m) contains more than 11.25 mol m^−3^ NaCl and the same amount of ethanol, and the density of the solution is greater than the density of water-filling compartments (l) and (r). An analogous mechanism for generating negative, zero and positive potential $\psi_{S}$ operates for solutions containing NaCl and 100 mol m^−3^ of ethanol and water.

In order to use the membrane potential model expressed by Equations (15) and (28) and calculate $∆\psi_{S}$, one must experimentally determine the dependence ${∆\tau }_{m}=f(C_{m}{C_{0}}^{-1})$, $∆\tau_{0}$, $\zeta_{r}^{B}=f(C_{m}{C_{0}}^{-1})$ and $\zeta_{l}^{B}=f(C_{m}{C_{0}}^{-1})$. The coefficients ${∆\tau }_{m}$ and $∆\tau_{0}$ were determined according to the methodology described in [16]. In turn, the values of the coefficients $\zeta_{r}^{B}$ and $\zeta_{l}^{B}$ were determined according to the methodology described in [15]. A single-membrane system was used to test ${∆\tau }_{m}$, $∆\tau_{0}$, $\zeta_{r}^{B}$ and $\zeta_{l}^{B}$. To convert the double-membrane system shown in Figure 2, vessel (m) was removed to the single-membrane system. In configuration *A* of this system, vessel (r) was filled with the solution under study and vessel (l) was filled with NaCl solution with a concentration of $C_{0n}$ = 1 mol m^−3^, while in configuration *B* the locations of the solutions were swapped.

**Figure 6:**
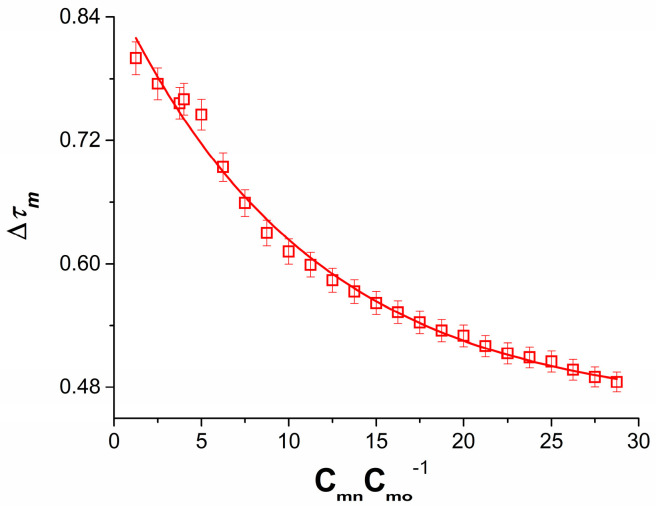
The dependence ${∆\tau }_{m}=f(C_{m}{C_{0}}^{-1})$ for NaCl solutions in 50 mol m^−3^ aqueous ethanol solution.

In Figure 6, which shows the dependence ${∆\tau }_{m}=f(C_{m}{C_{0}}^{-1})$, it can be seen that as the value of $C_{m}{C_{0}}^{-1}$ increases, the value of $\tau_{m}$ decreases exponentially. On the other hand, ${∆\tau }_{0}$ in the studied interval of $C_{m}{C_{0}}^{-1}$ is independent of concentration and is $∆\tau_{0}$ = 0.216 [16].

**Figure 7:**
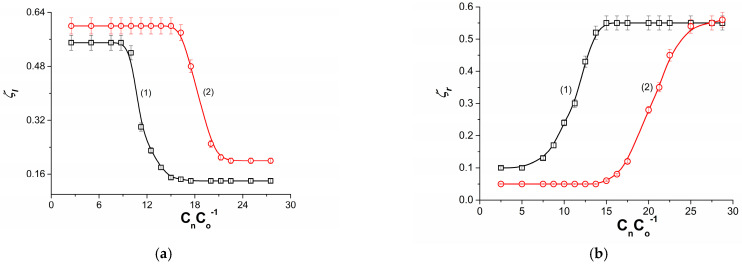
(**a**) Dependences $\zeta_{l}^{B}=f(C_{m}{C_{0}}^{-1})$ for aqueous solutions of NaCl and ethanol with $C_{me}$ = 50 mol m^−3^ (curve 1) and $C_{me}$ = 100 mol m^−3^ (curve 2). (**b**) Dependences $\zeta_{r}^{B}=f(C_{m}{C_{0}}^{-1})$ for aqueous solutions of NaCl and ethanol with $C_{me}$ = 50 mol m^−3^ (curve 1) and $C_{me}$ = 100 mol m^−3^ (curve 2).

Figure 7a shows the dependence $\zeta_{l}^{B}=f(C_{m}{C_{0}}^{-1})$ for aqueous NaCl solutions and fixed ethanol concentrations $C_{me}$ = 50 mol m^−3^ (curve 1) and $C_{me}$ = 100 mol m^−3^ (curve 2). In this case, the solution with the smaller and fixed concentration of NaCl is in the compartment above the membrane. In the compartment below the membrane is a solution with increasing NaCl concentration and a fixed ethanol concentration. It can be seen from these curves that, initially, despite the increase in $C_{m}{C_{0}}^{-1}$, the value of $\zeta_{l}^{B}$ is constant. In this region of NaCl concentrations, its contribution to the creation of solution densities is smaller than that of ethanol. This means that the $l_{l}^{B}$/M_l_/$l_{lm}^{B}$ complex is hydrodynamically unstable due to free convection. In the area where $\zeta_{l}^{B}$ decreases, the $l_{l}^{B}$/M_l_/$l_{lm}^{B}$ complex free convection gradually disappears and the $l_{l}^{B}$/M_l_/$l_{lm}^{B}$ complex stabilizes. In the region $C_{m}{C_{0}}^{-1}$, where $\zeta_{l}^{B}$ reaches a constant and minimum value, the $l_{l}^{B}$/M_l_/$l_{lm}^{B}$ complex is hydrodynamically stable due to the disappearance of natural convection and the presence of molecular diffusion.

**Figure 8:**
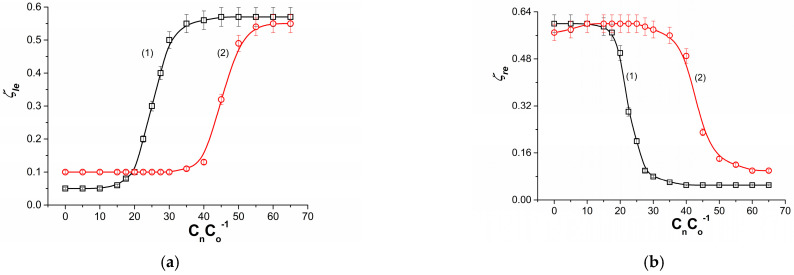
(**a**) Dependencies $\zeta_{le}^{B}=f(C_{me}{C_{0}}^{-1})$ for ethanol solutions in aqueous NaCl solution and with $C_{mn}{C_{0}}^{-1}$ = 5 (curve 1) and $C_{mn}{C_{0}}^{-1}$ = 10 (curve 2). (**b**) Dependencies of $\zeta_{re}^{B}=f(C_{me}{C_{0}}^{-1})$ for ethanol solutions in aqueous NaCl solution and with $C_{me}{C_{0}}^{-1}$ = 5 (curve 1) and $C_{mn}{C_{0}}^{-1}$ = 10 (curve 2).

Figure 7b shows the dependence $\zeta_{r}^{B}=f(C_{m}{C_{0}}^{-1})$ for aqueous NaCl solutions and fixed ethanol concentrations $C_{me}$ = 50 mol m^−3^ (curve 1) and $C_{me}$= 100 mol m^−3^ (curve 2). In this case, the solution with the smaller and fixed concentration of NaCl is in the compartment below the membrane. In the compartment above the membrane is a solution with increasing NaCl concentration and a fixed ethanol concentration. It can be seen from these curves that, initially, despite the increase in $C_{m}{C_{0}}^{-1}$, the value of $\zeta_{r}^{B}$ is constant and minimal. In this region of NaCl concentrations, its contribution to the creation of solution densities is smaller than that of ethanol. This means that the $l_{l}^{B}$/M_l_/$l_{lm}^{B}$ complex is hydrodynamically stable due to the absence of natural convection. In the area where $\zeta_{r}^{B}$ increases, the $l_{l}^{B}$/M_l_/$l_{lm}^{B}$ complex is destroyed due to increasing natural convection. In the area $C_{m}{C_{0}}^{-1}$, where $\zeta_{r}^{B}$ reaches a constant and maximum value, the $l_{l}^{B}$/M_l_/$l_{lm}^{B}$ complex is hydrodynamically unstable due to the disappearance of molecular diffusion and the maximization of natural convection. Incorporating the experimental results shown in Figure 6 and Figure 8a,b into Equations (15) and (28), curves 1, 2 and 3 shown in Figure 5a were obtained. They show good agreement between experimental and computational results.

To use the membrane potential model expressed by Equations (15) and (28) and calculate $∆\psi_{S}$, use the dependence ${∆\tau }_{m}=f(C_{m}{C_{0}}^{-1})$, shown in Figure 6, $∆\tau_{0}$ = 0.216, $\zeta_{r}^{B}=f(C_{me}{C_{0e}}^{-1})$ and $\zeta_{l}^{B}=f(C_{me}{C_{0e}}^{-1})$.

Figure 8a shows the dependence $\zeta_{l}^{B}=f(C_{me}{C_{0}}^{-1})$ for ethanol solutions in aqueous NaCl solution of aqueous NaCl solutions and fixed values of NaCl concentrations. Curve (1) was obtained for $C_{m}{C_{0}}^{-1}$ = 5 while curve (2) was obtained for $C_{m}{C_{0}}^{-1}$ = 10. In this case, a solution with a lower ethanol concentration and a fixed NaCl concentration is in the compartment above the membrane. In the compartment below the membrane is a solution with increasing ethanol concentration and fixed NaCl concentration. These curves show that, initially, despite the increase in ethanol, the value of $\zeta_{l}^{B}$ is constant and minimal. In this region of ethanol concentrations, its contribution to the creation of solution densities is smaller than that of NaCl. This means that the $l_{l}^{B}$/M_l_/$l_{lm}^{B}$ complex is hydrodynamically stable due to the barrack of natural convection. In the area where $\zeta_{l}^{B}$ increases, the $l_{l}^{B}$/M_l_/$l_{lm}^{B}$ complex gradually becomes unstable and natural convection gradually increases and the $l_{l}^{B}$/M_l_/$l_{lm}^{B}$ complex destabilizes. In the area $C_{m}{C_{0}}^{-1}$, where $\zeta_{l}^{B}$ reaches a constant and maximum value, the $l_{l}^{B}$/M_l_/$l_{lm}^{B}$ complex is hydrodynamically unstable due to intense natural convection.

Figure 8b shows the dependence $\zeta_{r}^{B}=f(C_{me}{C_{0e}}^{-1})$ for aqueous ethanol solutions. Curve (1) was obtained for NaCl with a fixed $C_{mn}{C_{0}}^{-1}$ = 5 (curve 1) and $C_{mn}{C_{0}}^{-1}$ = 10 (curve 2). In this case, the solution with a lower and constant NaCl concentration is in the compartment below the membrane. In the compartment above the membrane is a solution with increasing ethanol concentration and a fixed NaCl concentration. It can be seen from these curves that, initially, despite the increase in $C_{mn}{C_{0}}^{-1}$, the value of $\zeta_{r}^{B}$ is constant and maximum. In this region of ethanol concentrations, its contribution to the creation of solution densities is greater than that of ethanol. This means that the $l_{l}^{B}$/M_l_/$l_{lm}^{B}$ complex is hydrodynamically stable due to the absence of natural convection. In the area where $\zeta_{r}^{B}$ decreases, the $l_{l}^{B}$/M_l_/$l_{lm}^{B}$ complex gradually stabilizes due to decreasing natural convection. In the area where $C_{mn}{C_{0}}^{-1}$ reaches a constant and maximum value, the $l_{l}^{B}$/M_l_/$l_{lm}^{B}$ complex is hydrodynamically stable due to the disappearance of natural convection and the maximization of molecular diffusion. Incorporating the experimental results shown in Figure 6 and Figure 8a,b into Equations (15) and (28), curves 1 and 2 shown in Figure 5b were obtained. They show good agreement between experimental and computational results.

In real conditions, transport processes occur spontaneously. These processes are generated and regulated by different types of driving forces that participate in the creation of different types of physical fields (scalar, vector and tensor) that participate in shaping the field’s nature. A typical manifestation of a scalar field is the fields of concentrations, pressures, temperatures or electric potentials. The gravitational field, on the other hand, is a typical representation of a vector field. In addition to field creation, driving forces revealed through gradients of concentration, pressure, temperature and/or electric potential generate various types of transport, including membrane transport of volume, mass, energy and/or charge. Membrane transport efficiency is regulated by reducing the driving forces, such as the membrane concentration gradient. Under real conditions, the cause of this reduction is most often concentration polarization, the cause of which is molecular diffusion. A manifestation of concentration polarization is the creation of concentration boundary layers. Under the conditions of the Earth’s gravitational field, the membrane concentration gradient can be rebuilt. The reconstruction process is initiated and developed by hydrodynamic instabilities, the intensity of which even leads to the appearance of dissipative structures. 

In addition to areas of CBLs, hydrodynamic instabilities caused by the gravitational field also occur in nature in the form of convective motions in the atmosphere and oceans [32,33]. In the latter, saltwater density gradients appear in the vertical direction, caused by temperature differences or varying degrees of water salinity. When solution density gradients are reproduced due to external stimuli, such as temperature and/or concentration gradients on the membrane, there is a mutual “attrition” of these opposing forces leading to a cyclic strengthening and weakening of the convective flux. When the stimulus conditions reproducing the density gradients of the medium are established, such as establishing the temperatures of the two surfaces—the upper smaller and the lower larger, regular space–time structures known as Rayleigh–Benard convection cells are observed. The nature of these structures depends on the value of the Rayleigh number.

The graphical representation of these processes is provided by the temporal and concentration characteristics of membrane potentials, determined using the original measurement set. These characteristics are nonlinear and dependent on both the concentration and the composition and density of the solutions separated by the membranes. It is possible to choose solution concentrations and solution compositions so that the densities of the solutions located in the intermembrane compartment and in the outer compartments are identical. Then, the symmetry of the CBLs’ complexes results in the zeroing of the gravielectric effect. 

Knowledge of the mechanisms of generation of these phenomena can be important in considering biological systems, whose internal environment is aqueous solutions of various types of ions, heterogeneous due to the existing boundaries between different areas, both at the cellular and tissue levels. Solutions in biological systems are characterized by the presence of many types of ions and non-ionic substances, the gradients of which can cause gradients in the density of solutions. Through these structures appearing either spontaneously or intentionally, the gravitational field occurring as a constant near the Earth’s surface can affect cellular as well as tissue processes of biological systems. On the other hand, studies of biological systems under conditions of Earth’s gravity, in microgravity [8,34,35], show significant changes occurring in the structure and functioning of the biological system, which is probably caused by the disruption of the previously mentioned biological system processes. Therefore, the research can approximate the picture of potential changes in the heterogeneous structures and complex processes of biological systems caused by the gravitational field.

## 4. Conclusions

1. Initially, the double-membrane system is osmotically, diffusively and electrically symmetric under conditions of absence of concentration boundary layers (CBLs) and conditions of symmetric formation due to molecular diffusion of CBL complexes on both sides of each membrane. The system loses the symmetry of CBLs formation when hydrodynamic instabilities appear in the area of one of the complexes (non-convective state) leading, after exceeding the critical value of the concentration Rayleigh number, to a convective state.
2. Within the framework of the Kedem–Katchalsky formalism, an ion transport model was developed that includes membrane and solution transport parameters and takes into account hydrodynamic (convective) instabilities for a double-membrane system. It is shown that the transition from the non-convective state to the convective state or vice versa can be controlled by a dimensionless concentration polarization factor or concentration Rayleigh number.
3. The higher NaCl concentration in the solution causes the higher density of the solution in the intermembrane than in the outer compartment and induces convectional movements around the lower membrane. This entails the appearance of a positive gravielectric effect. In contrast, higher ethanol concentration in the solution causes a lower density of the solution in the intermembrane than in the outer compartment and induces convectional movements around the upper membrane, leading to a negative gravielectric effect. Such behavior of the double-membrane system indicates its regulator properties, due to its arbitrary switching from “−”, “0” or “+” states. The double-membrane electrochemical system considered in this paper is a source of electromotive force. In addition, the considered double-membrane system can be a model of an electrochemical gravireceptor.

## Data Availability

The datasets for this study are available upon request from the corresponding author.

## List of Symbols

M_l_, M_r_	membranes
CBLs	concentration boundary layers
r = *A* or *B*	configuration *A* or *B*
$l_{l}^{r}$/M_l_/$l_{lm}^{r}$	complexes membranes/CBLs in configuration *A* or *B*
$l_{mr}^{r}$/M_r_/$l_{r}^{r}$	complexes membranes/CBLs in configuration *A* or *B*
$l_{l}^{r}$, $l_{lm}^{r}$, $l_{rm}^{r}$, $l_{r}^{r}$	CBLs in configuration *A* or *B*
$\delta_{l}^{r}$, $\delta_{lm}^{r}$, $\delta_{rm}^{r}$, $\delta_{r}^{r}$	thickness of CBLs in configurations *A* or *B*
$C_{l}$$,\;C_{m}$,$C_{r}$	concentrations of solutions outside CBLs
$C_{l}^{r}$	concentration of solution at $l_{l}^{r}$/M_l_ boundary
$C_{ml}^{r}$	concentration of solution at $M_{l}/l_{lm}^{r}$ boundary
$C_{mr}^{r}$	concentration of the solution at the border of $l_{rm}^{r}$/M_r_
$C_{r}^{r}$	concentration of the solution at the border of $M_{r}/l_{r}^{r}$
$R_{c}$	concentration Rayleigh number
$J_{v}$	volume flux (m s^−1^);
$J_{s}$	solute flux (mol m^−2^s^−1^)
$I_{q}$	electric charge flux (A)
$L_{p}$	hydraulic conductivity coefficient (m^3^ N^−1^s^−1^
σ	reflection coefficient
$P_{E}$	electroosmotic permeability coefficient (N A^−1^)
ω	solute permeability coefficient (mol N^−1^s^−1^)
γ	Van’t Hoff coefficient
*R*	gas constant (J mol^−1^K^−1^)
*T*	absolute temperature (K)
κ	electrical conductivity (Ω^−1^m^−2^)
$t_{a}$, $\tau_{c}$	transfer number of anion (a) and cation (c)
$\nu_{j}$	ion number
$z_{j}$	valence
*F*	Faraday’s constant. (C mol^−1^)
$\bar{C}$	average concentration of the solution (mol m^−3^)
$∆\psi_{m}$	potential difference measured with two reversible electrodes (V)
$∆\psi_{l}^{r}$, $∆\psi_{r}^{r}$	electrical potential difference across the $l_{l}^{r}$ and $l_{r}^{r}$ layers (V)
$∆\psi_{Ml}^{r}$, $∆\psi_{Mr}^{r}$	electrical potential difference across the M_l_ and M_r_ membranes (V)
$∆\psi_{lm}^{r}$, $∆\psi_{rm}^{r}$,	electrical potential difference across the $l_{lm}^{r}$ and $l_{rm}^{r}$ layers (V)
$I_{l}^{r}$, $I_{r}^{r}$	ionic current through layer $l_{l}^{r}$ and $l_{r}^{r}$ (A)
$I_{Ml}^{r}$, $I_{Mr}^{r}$	ionic current through membrane M_l_ and M_r_ (A)
$I_{lm}^{r}$	ionic current through layer $l_{lm}^{A}$ (mol m^−2^s^−1^)
$J_{l}^{B}$	soute flux through $l_{l}^{B}$ layer (mol m^−2^s^−1^)
$J_{sl}^{B}$	solute flux through $l_{l}^{B}$/M_l_/$l_{lm}^{B}$ complex (mol m^−2^s^−1^)
$J_{ml}^{B}$	solute flux through $l_{lm}^{B}$ layer (mol m^−2^s^−1^)
$J_{vl}^{B}$	volume flux through $l_{l}^{B}$ layer (m s^−1^)
$J_{vsl}^{B}$	volume flux through $l_{l}^{B}$/M_l_/$l_{lm}^{B}$ complex (m s^−1^)
$J_{vml}^{B}$	volume flux through $l_{lm}^{B}$ layer (m s^−1^)
